# Risk–benefit assessment of foods and its role to inform policy decisions: outcome of an international workshop

**DOI:** 10.3389/fnut.2024.1458531

**Published:** 2024-09-25

**Authors:** Constanza De Matteu Monteiro, Jeanne-Marie Membré, Morten Poulsen, Sofie Theresa Thomsen, Sara Monteiro Pires

**Affiliations:** ^1^National Food Institute, Technical University of Denmark, Kongens Lyngby, Denmark; ^2^Oniris VetAgroSup, INRAE, Secalim, Nantes, France

**Keywords:** risk–benefit assessment, food policy, decision-making, health impact assessment, holistic approaches

## Abstract

Policy decisions in public health require consideration and evaluation of trade-offs for which transparency and science-based evidence is needed. Improvement of decision-support tools is essential to help guide food policy decisions that promote healthy diets and meet the challenges of food systems without compromising food security, food safety, and sovereignty. Risk–benefit assessment of foods (RBA) is an established methodological approach designed to inform policy decisions within the area of nutrition and food safety. Despite methodological developments, translation of RBA findings into policies is still limited. In this context, a stakeholder workshop held in May 2023 gathered RBA experts and food regulators from Europe to identify the challenges, obstacles and opportunities in using evidence generated through RBAs to inform food policy decisions. A structured process was implemented to collect their views through online surveys, breakout groups, and plenary discussions. As a secondary objective, food regulators’ views on other approaches for holistic risk assessment fit for food systems analysis were also explored. This paper summarizes the main findings of the workshop and discusses policy implications and future perspectives to improve the area of RBA and its role in food policymaking.

## 1 Introduction

Governance targeting healthy and safe diets has been a central part of international strategies to reduce the burden of communicable and noncommunicable diseases (1, 2). As dietary habits are still among the leading behavioral risks factors contributing to global mortality, strengthening food policies and public health actions related to dietary choices remains crucial to reduce the burden of disease of populations (3, 4). Since these public health policies need to be prioritized to tackle the most important risk factors, while ensuring that food safety risks are not introduced, there is an increasing need for decision-support tools that are able to evaluate the health impact of diets and food systems considering both nutrition and food safety (5, 6).

Risk–benefit assessment (RBA) of foods is a decision-support tool that estimates the public health impact of foods and diets by evaluating both beneficial and adverse health effects in different exposure (e.g., often consumption) scenarios (3, 7). The evidence generated in RBAs aims to support priority-setting and formulation of policies that are coherently aligned across several disciplines (i.e., nutrition, toxicology, and microbiology) (8, 9). RBA builds on the risk assessment framework by mirroring its four steps (i.e., hazard identification, hazard characterization, exposure assessment and risk characterization) in a parallel assessment for beneficial effects (10, 11).

RBA and its methodologies have evolved over the past decades (3, 5, 7). Several case-studies and activities for capacity building for RBAs have been conducted within many research projects financed by the European Union (EU) (7–10, 12, 13). These case studies predominantly assessed the health impacts of scenarios of consumption of specific foods (e.g., fish and seafood; nuts; rice) (14–16); of food substitutions (e.g., meat for fish; meat for pulses) (17–19) including substitution scenarios with novel foods (20); or individual food components (e.g., iodine; folic acid) (21, 22). These studies have also led to an increased interest in RBA by the scientific community, and a growing body of evidence in risk–benefit relations of different foods and dietary patterns in populations across the EU. Furthermore, RBAs have been adopted by several food authorities including the European Food Safety Authority (EFSA), which recently updated their guidance on human health RBA, firstly published in 2010 (23–25). Despite this broad interest, these activities have not been accompanied by timely translation of knowledge into policies. Thus, there is a need for unraveling the potential of RBAs and increasing its visibility among regulatory bodies to ensure a wider application in policy making settings. If links between RBAs, risk–benefit management decisions, and communication of dietary recommendations are strengthened, more transparency and effective public health actions related to dietary choices could potentially be achieved (5, 26). This paper contributes to the limited literature that discusses the role of RBA and the gaps hindering its practical applications into policy decisions related to foods.

The HOLiFOOD project, a four-year research project (2022–2026) funded by the European Commission under the Horizon Europe Program and aiming to introduce a holistic approach for tackling food systems risks in a changing global environment (27), gathered a group of RBA experts and food regulators for an international workshop. The main objective of the workshop was to identify the challenges, obstacles, and opportunities in using evidence generated through RBAs to inform food policy decisions in the European context. Since RBAs could be an adaptable tool for food system analysis and useful to inform potential impacts of dietary shifts caused by different drivers such as sustainability and climate change, stakeholders´ views on the broader applications of RBAs were also briefly investigated. Hence, secondary objectives of the workshop were: (i) to investigate to which extent food regulators were aware or previously used output from RBA to support regulatory tasks related to public health in food safety and nutrition; and (ii) to explore food regulators’ views on other approaches for holistic assessment, defined as the integrated assessment of health and sustainability impacts of food systems. This paper summarizes the main findings of the workshop, contributing with the yet emergent and novel debate on the implications and future perspectives of RBA for an enhanced role in food policy.

## 2 Methods

### 2.1 Workshop structure

The stakeholder workshop “Health Risk–Benefit Assessments: from Science to decision-making” was held online in May 2023, with a cohort of participants consisting of risk–benefit assessors, managers, and communicators. A structured process was implemented to gather the views of experts in RBA, experts in risk (and benefit) communication, and food policymakers through online surveys, group and plenary discussions.

An initial pool of participants was created based on the networks of the HOLiFOOD consortium members and by searches of relevant food authorities across different EU Member States. The individual people contacted were free to redirect or expand the invitation of the workshop with their coworkers if they wished so. Participation in the workshop was voluntary and followed the EU General Data Protection Regulation (GDPR) enforced in the HOLiFOOD project. The approach applied to engage with participants was structured in four steps: anonymous voluntary surveys (prior and during the workshop); an introductory keynote presentation; breakout groups with guided discussion points; and a final moderated plenary discussion.

### 2.2 Pre-workshop and in-workshop surveys

Invitations to participate in the pre-workshop survey were sent out to the invitees that confirmed interest and availability to attend the meeting approximately 1 month prior to the event. The pre-workshop survey which was supported by the SurveyXact platform,^1^ served to tailor the workshop content and query the invited participants about any potential discussion points that were expected to be covered during the meeting, besides collecting information on the participants’ background, expertise, and level of knowledge of RBA. During the workshop, the collaborative online tool Mentimeter^2^ was applied to collect and display to the group the participants’ background, level of knowledge, and experience on RBA, as well as to address the secondary objectives of the workshop by collecting their views on the need for RBA approaches that consider non-health dimensions in RBA. The tool was used prior to the breakout groups and at the end of the workshop. The audience’s response was displayed to all participants and served as feedback and prompt to start discussions. The questionnaires are available in the Supplementary material.

### 2.3 Break-out groups and plenary discussions

During the discussion sessions, participants were invited to reflect on previous experiences on RBA application or usage of results and the information presented by the keynote speaker, and to contribute to moderated discussions on the following topics:

*Theme 1*: Challenges of using RBAs to inform food-related policy decisions (e.g., could challenges be related to the structural organization of authorities?).

*Theme 2*: Opportunities and needs concerning RBAs (e.g., could challenges be related to the reliability of the RBA methods?).

*Theme 3*: Communication of RBAs (e.g., could challenges be related to how the results are communicated?).

**Figure 1:**
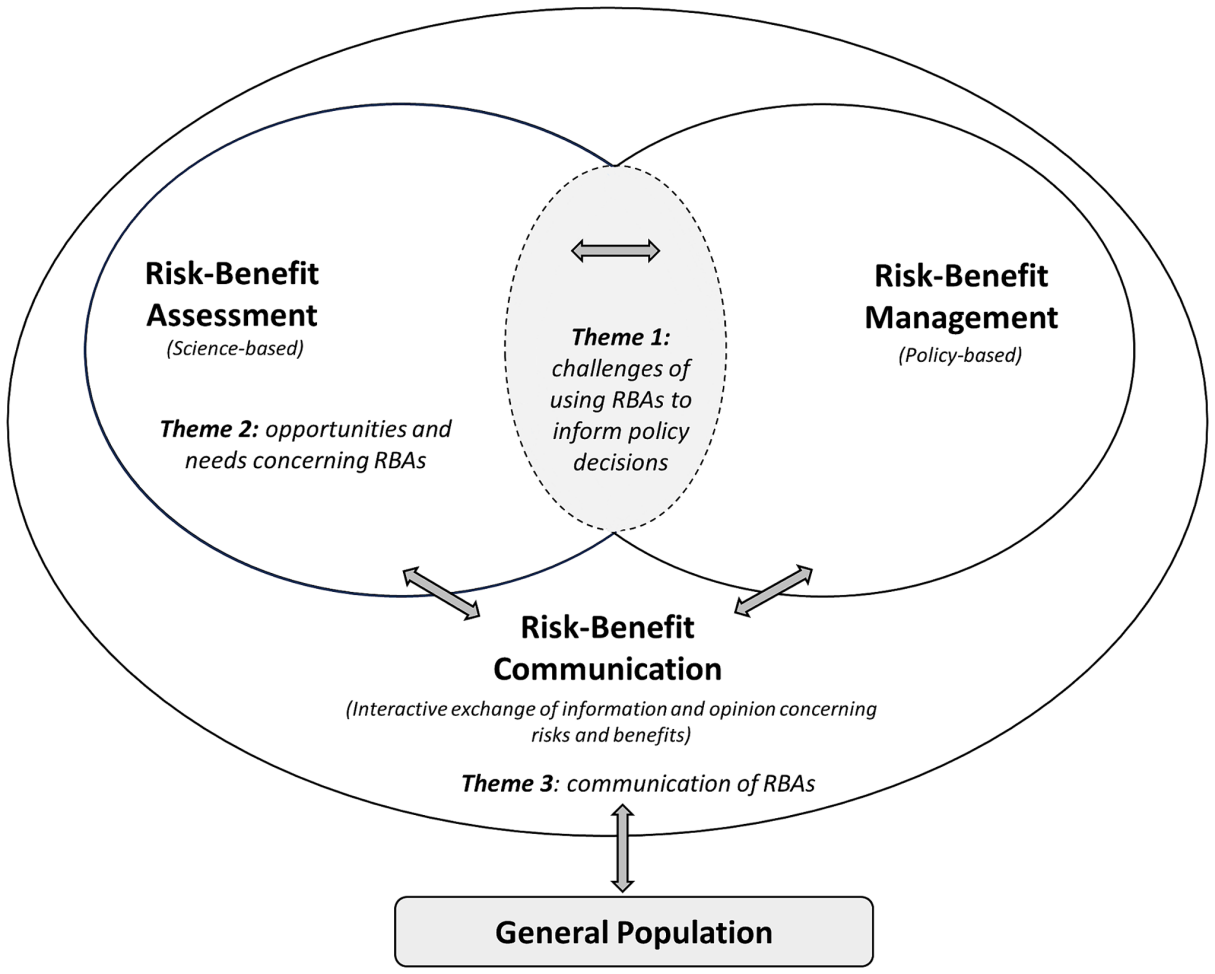
The risk–benefit analysis paradigm and the discussions’ themes of the workshop. Adapted from Nauta et al., licensed under CC BY 4.0 (8).

The selected themes were associated with one or more components of the risk–benefit analysis paradigm (Figure 1), as proposed by Nauta et al. (8). Participants were divided into three groups. The workshop’s facilitators ensured that each group had a similar number of participants with diverse backgrounds, and that all breakout groups discussed the three themes. During the breakout session, participants were invited to express their views at will. The information collected during the workshop was captured by three different rapporteurs and video recording.

In plenary, rapporteurs of each breakout group summarized the key discussion points, followed by the moderated discussion at plenum. After the information was extracted for analysis and cross-checked, the video recording was deleted.

## 3 Results

In total, 17 anonymous volunteers participated in the pre-workshop survey. Respondents suggested a variety of topics to be addressed in the workshop, ranging from questions on data requirements for RBA and methodological considerations to possible expansion of RBA approaches beyond health concerns (Table 1). All proposed topics were included as discussion points in the workshop. Due to time constraints, the suggested topics that were not specific to the health dimension were only addressed more broadly during the session on holistic approaches.

**Table 1:** Discussion points related to risk–benefit assessment (RBA) of foods suggested by participants in the pre-workshop survey.

Discussion points suggested by respondents
What is the type of data needed and minimum requirements? How to compare different risks or benefits, and in which scale or metric? Uncertainties in RBA Selection of health components Real-life examples of how risk–benefit studies have managed to reach policymakers With exception of fish and seafood products, for which other food categories would RBA be useful Systematic approaches to handle uncertainties in RBAs Shortcoming of the RBA models Ways to communicate the results of RBAs to the public Is performing RBAs the responsibility of risk assessors or risk managers? How to quantify and rank risks when different health outcomes (chronic and acute) are considered together Could RBAs be more informative to risk managers if it was not exclusively centered on human health? Have more comprehensive RBAs (addressing multiple contaminants in foods) RBAs and socio-economics issues

About half of the invitees confirmed both interest and availability to contribute to the workshop (initial pool of participants were approximately 50 people). In total, the stakeholder workshop gathered 37 participants from 19 institutions across 13 countries (see Appendix). The initial level of familiarity with RBA varied. Most of the participants had prior knowledge of RBAs, as self-stated in the surveys (familiar with RBAs, *n* = 10; some general knowledge on RBAs, *n* = 7; limited to no RBA knowledge, *n* = 6; preferred not to answer, *n* = 14).

**Figure 2:**
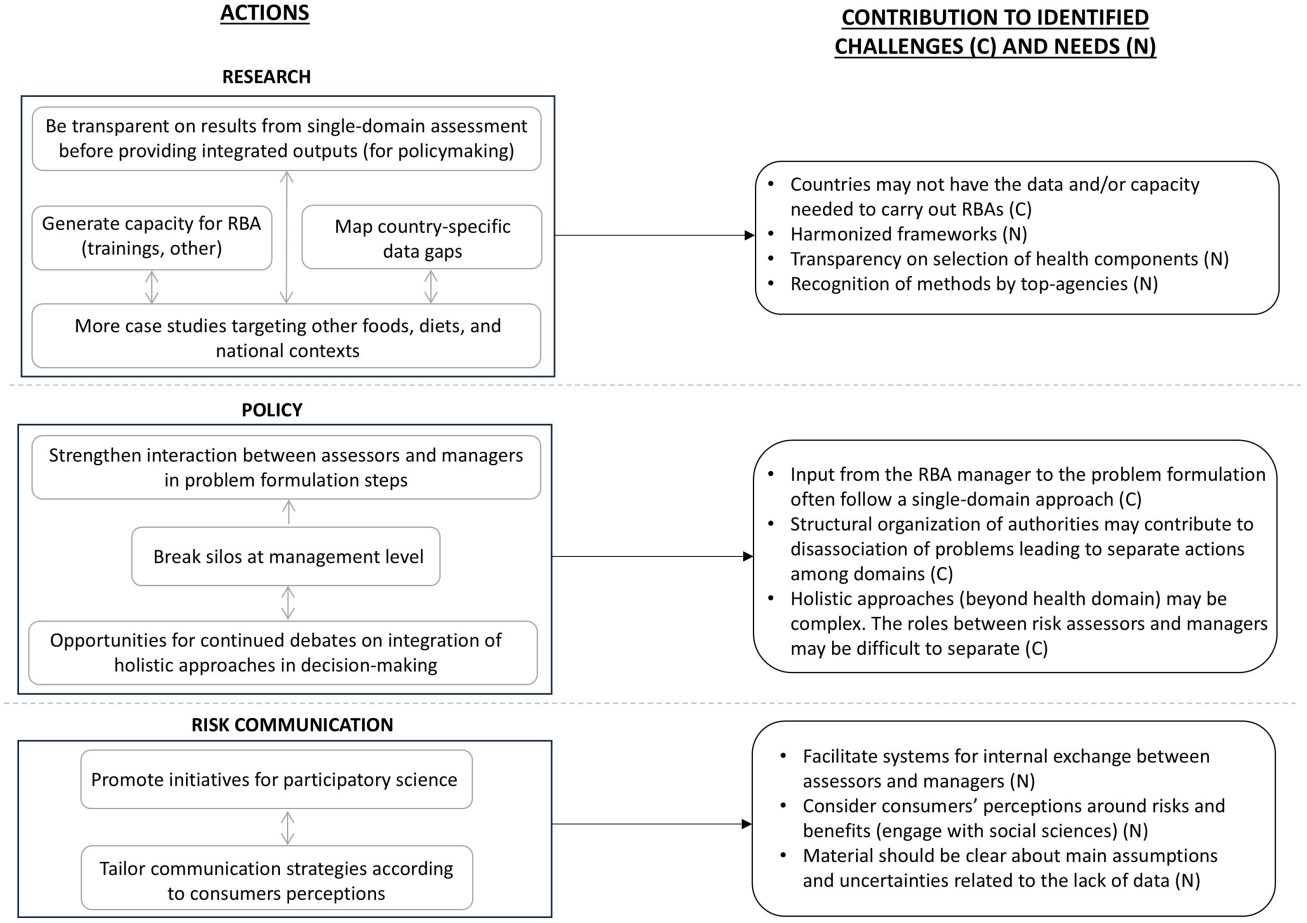
Summary of suggested actions to increase the adoption of risk–benefit assessment (RBA) for food-related policies and contributions of the identified challenges and needs following the findings of the international stakeholder workshop.

During the workshop, either in plenary or in the breakout groups, participants shared examples of relevant RBA cases conducted in their country (e.g., on fish consumption or to inform recommendations on consumption of nuts), and exchanged lessons learned in their countries when communicating findings or using outputs from RBAs to support regulatory tasks. Additionally, discussion points brought up by participants and covering the themes previously introduced are presented below. A summary of main actions addressing the challenges, needs, and opportunities identified in the workshop and clustered by the authors are presented in Figure 2.

### 3.1 Challenges of using RBAs to inform food-related policy decisions

This theme identified and discussed challenges of using RBAs to inform food-related policy decisions. Participants recognized that, in countries across the EU, food safety and nutrition are traditionally separated domains, which is also reflected in the structural organization of food authorities. Consequently, it was argued that this division between nutrition and food safety departments may determine the type of questions posed by policymakers to risk (and benefit) assessors, and thus impact on the type of evidence being generated. It was highlighted that this “dissociation” of decision-making problems may lead to processes, priorities, and evidence being used to inform food policy decisions to result in separate actions within each of these fields. Therefore, requests for evidence formulated “in silos” was identified as one possible obstacle to addressing problems in a multidisciplinary approach as well as to promoting multisectoral actions across food safety, nutrition, and potentially sustainability. In this context, strengthening the communication between risk–benefit assessor and manager, alongside with multidisciplinary collaboration at risk management level could be considered as important elements for improving the formulation of decision-making problems.

In terms of collaboration between food safety and nutrition departments for RBAs, both successful and challenging examples at national level were reported. In one of the examples, disentangling interests to communicate outputs that translates both risks and benefits in an equal manner was reported as difficult, especially if external stakeholders were involved.

For the subtheme on holistic approaches, participants highlighted the need for future assessments to appropriately account for sustainability factors. Although RBAs could serve as a stepping stone for developing methods to assess the multi-dimensional impacts of foods by taking a food systems approach, several challenges linked to holistic approaches were discussed. For example, including other dimensions beyond health in RBAs might make the assessment resemble a decision-making process, as opposed to a process that provides evidence for decision-making. This can be problematic as the roles between risk assessors and managers will no longer be clearly defined. Furthermore, integrating other dimensions such as economic and environmental factors may increase the complexity and resources, including data, needed for the assessment. This could also increase the uncertainty introduced in the results and complicate the communication of outputs. Concerns in relation to the potential loss of information and transparency when dimensions are integrated were also expressed. Policy makers should be able to discern and navigate through the results of assessments from the micro (i.e., each dimension) and macro (i.e., integrated dimensions) perspective. In summary, it was suggested to run individual (i.e., single dimension) assessments before integration into one metric or output.

Lastly, an important challenge hindering the adoption of RBAs at a larger scale and internationally is that many countries still have neither the data nor the capacity needed to carry out RBAs. Hence, a clear actionable point highlighted was to continue supporting initiatives to build capacity within RBAs, as well as mapping country-specific data gaps and making data accessible.

### 3.2 Opportunities and needs concerning RBAs

This theme aimed at identifying ways to overcome obstacles related to the acceptability of RBA methods. Participants identified a variety of methodological, communication and awareness-raising needs to enhance the use of RBA outputs for regulatory decisions. They also acknowledged opportunities to address some of these needs. Opportunities and needs are summarized and presented in Table 2.

**Table 2:** Summary of opportunities and needs for risk–benefit assessment (RBA) development identified by participants of the stakeholder workshop.

Needs	Opportunities
Simplified RBA approaches, which should be presented as a less complex, resource-demanding and time-consuming calculations.	
Harmonized frameworks as assessments considering different beneficial and adverse effects while responding to similar risk–benefit questions might generate different advice.	Development of more RBA case-studies through research projects. Development of harmonized frameworks and methodologies for RBA that can be applied by national research institutions.
Transparency in communication of approaches, data used, model assumptions, and intermediate and final outputs of RBAs. Consumer trust might be diminished if advice from different assessments differ, and if transparent documentation and explanations are not provided.	
Objective and transparent framework on how the components to be included in the assessments are selected to ensure reproducibility.	
Harmonized processes to weigh the strength of available scientific evidence used to inform RBA and select data based on established criteria.	Accumulated experiences within RBA can support guidelines and ensure communication of methods, results and underlying uncertainties targeted at different stakeholders (scientists, risk managers, citizens, other stakeholders).
Increased number of case studies, tackling different foods, food components and diets, in different populations and countries.	Promote training activities to increase capacity for RBA within national and international institutions. Engagement with stakeholders at national and international levels can increase the interest of risk managers to formulate risk–benefit questions and allocate resources for RBAs.
Enhanced recognition of the utility and relevance of RBA by top agencies (e.g., WHO, FAO, etc.).	Seek more engagement and active contribution of international agencies where RBA activities have been already introduced (WHO/FAO, EFSA) for the development and applications of RBA case studies (25, 49).

Discussion in this theme emphasized that the selection of health components to be included in the model should be guided by objective criteria and a structured review of available scientific evidence and evaluation of its strength. However, time and resources do not always allow for a systematic review of the evidence, which may lead to biased choices in the selection of evidence and data used in the RBA. Furthermore, the lack of data to characterize risks and/or benefits may lead to incomplete assessments, an issue to which traditional health risk assessment is also subjected. Some participants noted that integrating risks and benefits in a balanced way is also challenging because risks, in comparison to benefits, are continuously evolving, with new contaminants often being discovered and assessed.

Finally, the expansion of RBA across countries and operationalization of RBA at global scale was discussed. Nevertheless, it can be argued that an RBA focused on a specific region or country is often more informative due to national and regional differences in, e.g., dietary habits, nutrient intakes, and contamination levels. Data reflecting variability in these factors could also lead to lower consumer trust if different RBAs on the same food yield divergent advice. As in risk assessment, this can be justified by the fact that RBA case studies are highly dependent on the data used and the populational context.

### 3.3 Communicating RBAs

Communicating both risk and benefits to citizens is important to ensure that dietary recommendations and trade-offs linked to dietary choices can be better understood. It was emphasized that the communication materials targeted to consumers should be clear about the fact that people are always protected by regulatory food safety frameworks. Some participants stressed that although food safety is never to be compromised, it is also relevant to demonstrate to consumers that some risks may be acceptable trade-offs for benefits. In addition, it was identified that to improve communication of RBA outputs, the communication materials and tools used to target policy-maker need to be different from those targeting citizens. Particularly for citizens, risk–benefit communication can have significant gains if investing in dialogs with the public, especially in understanding consumers´ perceptions around risks and benefits. To achieve this, expertise in social sciences is essential to help formulate appropriate communication strategies targeting consumers. Moreover, whether the target is citizens or food regulators, communication of RBA needs to include the assumptions and uncertainties of the approach, in addition to the main findings.

## 4 Discussion

To date, most of the publications on RBA have focused on the developments and future directions of the methodological framework, including articles reporting results of case-studies. Some authors have reviewed the different types of RBA studies (28–31), showing that most published case-studies have predominantly been conducted in the European context. Yet, to the best of our knowledge, no publication has tackled the bottlenecks in the practical application of the RBA findings to inform food-related policies. As the interaction and communication between risk–benefit assessors and regulators is of the utmost importance, the workshop outcome is regarded as a valuable contribution to the further development and implementation of RBA.

During the workshop, participants identified a variety of obstacles to using RBA outputs to inform regulatory decisions. These current obstacles explain the still limited translation of RBA findings into food-related policies and need to be addressed to ensure that decision maker can use this type of evidence that integrates knowledge from the multiple disciplines relevant to food systems. The workshop highlighted challenges, needs, and opportunities for RBAs that may be translated into tangible actions to further advance in this field. Although the online stakeholder workshop was short (less than half day program), the inputs reflected the diversity in background and geography of the participants and are helpful to guide current processes and next actions within RBAs.

Despite several methodological achievements, harmonization of RBA frameworks and simplified approaches are needed. Many of the RBAs carried out to date focused on fish and fish products (28–30). Thus, expanding the body of evidence with more case studies that target other foods or diets is important to further demonstrate the applicability of RBA. The experience from additional case studies could be beneficial to tackle obstacles that are interlinked as identified in the workshop (Figure 2). For example, it will help demonstrate the flexibility of the methods, contribute to the identification of data gaps, increase capacity building, provide further inputs for discussions that aim at harmonizing frameworks at international level, and explore ways to improve risk–benefit communication strategies.

A recent study from Boué and colleagues proposed a harmonized strategy to select health outcomes to be included in RBAs (32), resulting in a higher transparency of the selection process. This strategy is based on extensive literature searches, where a long list of components is created, contemplating in equal importance components relevant in nutrition, microbiology, and toxicology domains. This framework is divided into two steps for identifying, evaluating the strength of evidence, and selecting health outcomes based on defined criteria. This approach implies that if a health component is relevant for the RBA but is not included due to limited evidence, it is recommended that data gaps are communicated (32). Similar systematic approaches could be a starting point to enhance transparency on the selection of health components, a need for improvement in RBAs as identified in the workshop. A downside of this approach is that reviewing the literature can be time-consuming, and it is not always possible to conduct a systematic review prior to starting an RBA. This approach may also not be robust enough to capture emergent risks if potential new hazards are not identified in the literature review step or if not part of the risk–benefit question commissioned.

Nonetheless, if reporting on the scoping process of an RBA becomes a common practice among publications, actions to tackle previously identified data gaps could likely be facilitated.

Beside RBA, there are other methods to rank risks of food-related hazards that are also useful to inform food policy decisions (33). For example, based on an FAO guidance on informed decision-making considering multiple factors (34), a study adopted a Multi-Criteria Decision Analysis (MCDA) framework to rank risks from ready-to-eat dishes based on their nutritional, chemical and microbiological hazards (35). Even if the discussion of other methods is not in the scope of this paper, we emphasize the importance of understanding the strengths and limitations of RBAs as well as the type of questions RBAs can help informing so that methods chosen to inform a decision-making problem are fit-for-purpose.

Cross-departmental collaboration at risk–benefit assessor and management level were important elements discussed in the workshop and ways to strengthen partnerships are to be explored. As defining the decision-problem is the first step in health assessments, facilitating inclusion of both food safety and nutritional entities at regulatory level could facilitate the generation and applicability of integrated evidence. Better formulation of decision-making problems could trigger further developments and innovation in current working approaches. This could guide policies that are needed to handle multifaced problems.

Findings of the workshop also give insights for improvement of communication strategies for RBAs. In addition to being transparent on assumptions and uncertainties surrounding the data (or lack of it), participants pointed out the importance of involving social sciences in the development of communication strategies for RBAs. Promoting spaces for exchange and close dialog among researchers, food regulators and citizens is essential as it may help both in early assessment stages (e.g., to set up relevant and well-defined scope for cases studies), and in knowledge translation approaches for research dissemination. A recent review demonstrated the importance of an individual’s values and beliefs when purchasing foods (36). For example, in the European market, it was observed that consumers tend to give more importance to chemical risks (e.g., pesticides) than naturally occurring risks (36). The authors also demonstrated that risk acceptability in the population might differ based on the food item, and that understanding consumers´ perceptions on risks and benefits could be a way to tailor and improve communication materials of RBA findings targeted to citizens (36, 37). Furthermore, exploration of appropriated communication channels in relation to media and technological evolution should also be considered (38).

Several recent studies have quantified the negative environmental impact of diets and extensive efforts have been put to ensure that food policies and dietary recommendations are aligned to promote sustainable food systems (39–45). In this context, discussing holistic approaches that can assess the impact of diets and food systems beyond the health domain is extremely relevant (46–48). Due to the multidisciplinary character of the RBAs, participants’ views on expanding RBAs to become part of a broader food system analysis were briefly explored in the workshop, as previously proposed in the literature (5).

Moving toward holistic approaches would amplify some of the challenges related to data availability and the integration of different sources of data, increasing the uncertainty in the results and adding complexity in interpreting and communicating outputs. It is important to highlight that holistic approaches do not substitute the value and inputs provided by single domain or dimension assessment but rather inform different types of research questions and decision-making problems. Moreover, holistic approaches could improve transparency about the integration of different lines of evidence and application of outputs in public health policy decisions. Nevertheless, some contributions suggest that the integration of dimensions that involves value-based judgments should be rather conducted by risk–benefit managers.

Given that RBA is a multidisciplinary method, the workshop methodology allowed for more than one member per organization, especially if participants had different fields of expertise and worked in different organizational units. Although the breakout groups were designed to split stakeholders with similar scientific or organizational background, the authors acknowledge this as a main limitation, as the outcomes of the workshop could be subject to potential bias due to the selection and composition of the cohort of participants.

The input from stakeholders and outputs of the workshop demonstrates the need for the RBA community to continue an open dialog and exchange with food regulators for a more thorough discussion on the points raised in this theme. Future opportunities for exchange on RBA translation into policy settings should focus on expanding the topics presented in this work, engaging as well with a larger panel from scientists and regulators from other continents.

## 5 Conclusion

Stakeholders identified a wide range of needs, opportunities, and challenges to increase the use of RBA to inform food policy decisions. Despite diverse views, RBAs were unanimously acknowledged as a useful tool to generate dietary recommendations, including tailored advice to vulnerable groups of the population, and as a more transparent approach for consumers to understand potential trade-offs among certain dietary choices. While finding single solutions and reaching group consensus to the several obstacles identified were not in the scope of the workshop, main actions to enhance the role of RBA in policymaking as suggested by participants included: (i) developing harmonized approaches, strengthening capacity, and improving communication on RBA outputs, underlying limitations, and uncertainties; and (ii) working toward breaking silos between different disciplines, stakeholders, and risk–benefit assessors and managers.

## Data Availability

The raw data supporting the conclusions of this article is available upon request to the authors.

## Footnotes

^1^https://rambollxact.com

^2^www.mentimeter.com/

## Appendix

List of organizations contributing to the workshop.

**Table:**

Organization	Number of participants
ANSES—French Agency for Food, Environmental and Occupational Health & Safety	1
ASAE—Portuguese Economic and Food Safety Authority	1
BfR—German Federal Institute for Risk Assessment	7
DTU—Technical University of Denmark*	7
EC—European Commission (DG SANTE)	1
EFET—Hellenic Food Authority	2
EFSA—European Food Safety Authority	1
FAO—Food and Agricultural Organization of the United Nations	2
FCNAUP—Faculty of Nutrition and Food Sciences from University of Porto	2
FVST—Danish Veterinary and Food Administration	1
Hungarian Ministry of Agriculture	1
INRAE—French National Research Institute for Agriculture, Food and Environment*	1
NVWA—Netherlands Food and Consumer Product Safety Authority	1
Norwegian Food Safety Authority	1
SLV—Swedish Food Agency	2
The Dutch Ministry of Health, Welfare and Sport	1
UVMB—University of Veterinary Medicine Budapest*	1
UNEW—Newcastle University*	2
WHO—World Health Organization	2
*Workshop organizers and/or HOLiFOOD partners	
