# Supplementary material for: Risk–benefit assessment of foods and its role to inform policy decisions: outcome of an international workshop
Source: Front Nutr. 2024 Sep 25;11:1458531. doi: 10.3389/fnut.2024.1458531 (PMC11462872; doi:10.3389/fnut.2024.1458531)
Supplement: Supplementary file 1 [file Table_1.DOCX]

*Preworkshop survey*

*(supported by SurveyXact)*

*Question:* **What category represents your main working area?** *(multiple choice)*

( ) Food regulator (risk management)

( ) Risk communication

( ) Other *(open box)*

*Question:* **What is your main area(s) of expertise?** *(multiple choice)*

( ) Nutrition

( ) Toxicology

( ) Microbiology

( ) Epidemiology

( ) Biology

( ) Chemistry

( ) Medicine

( ) Science communication

( ) Other *(open box)*

*Question:* **Have you ever used outputs from RBAs of foods to support your work?** *(single choice)*

( ) Yes

( ) No

( ) Unsure

*Question:* **Do you think RBAs of foods is an applicable method for generation of evidence to support decision-making?** *(single choice – open question)*

( ) Yes – Please elaborate why *(open box)*

( ) No – Please elaborate why *(open box)*

*Question:* **In your opinion, is there any topic related to RBAs that should be addressed and discussed in the workshop?** *(open question)*

*(open box)*

*In-workshop survey*

*(supported by Mentimeter)*

**Round 1**

*Question:* ***How would you describe your current knowledge on risk-benefit assessment?*** *(single choice)*

( ) Limited to none. I am looking forward to learning more today

( ) I have some general knowledge on RBAs

( ) I am familiar with the RBA methods

*Question:* ***What category best represents your main working area?*** *(single choice)*

( ) Risk assessor

( ) Risk manager

( ) Risk communicator

( ) Other

**Round 2**

*Question:* ***Are you familiar with other holistic assessment methodologies? If yes, which?*** *(open question)*

*(open box)*
